# Comparison of characteristics and outcomes of premenopausal and postmenopausal women with adnexal torsion

**DOI:** 10.1177/03000605241305251

**Published:** 2024-12-23

**Authors:** MengHui Li, Hong Wang, ShengDi Hou, ShuZhen Wang, Hua Li

**Affiliations:** 1 Department of Obstetrics and Gynecology, Beijing ChaoYang Hospital affiliated to Capital University, China

**Keywords:** Adnexal torsion, premenopause, postmenopause, histological outcome, abdominal pain, nausea, vomiting

## Abstract

**Objective:**

This study aimed to compare the clinical characteristics and surgical and histological outcomes of premenopausal and postmenopausal patients with adnexal torsion.

**Methods:**

The electronic medical records of 278 patients with adnexal torsion proven by surgery were retrospectively reviewed from January 2012 to November 2023 in our hospital. The patients were divided into two groups (premenopausal and postmenopausal).

**Results:**

The study included 226 (81.3%) premenopausal patients and 52 (18.7%) postmenopausal patients. The incidence of the most common symptoms (i.e., abdominal pain, nausea and/or vomiting) was not different between the two groups. However, the postmenopausal group had a longer interval from the onset of pain to admission, a larger size of adnexal mass, a longer operation time, more blood loss, and a longer hospital stay than the premenopausal group. Regarding the procedure, the premenopausal group underwent more conservative procedures than the postmenopausal group. The most common pathological findings in the two groups were benign tumors and tubal pathology The malignancy rate was similar in the two groups.

**Conclusions:**

Premenopausal and postmenopausal women with adnexal torsion had similar main symptoms, such as abdominal pain and nausea and vomiting. However, the surgical and histological outcomes varied between these groups of women.

## Introduction

Adnexal torsion, which is a gynecological emergency, includes ovarian torsion, fallopian tube or para-tubal cyst torsion, or a combination of these conditions.^1^ Adnexal torsion is defined as the partial or complete twisting of the suspensory ligament that supports the blood supply to the ovary, and it can result in ovarian loss, infertility, and death if the treatment is delayed as a result of uncertainty in the diagnosis.^2,3^

Adnexal torsion can occur at all ages. The incidence of adnexal torsion varies in different studies and in different populations,^4^ but its peak prevalence is in women of reproductive age.^5^ The spectrum of clinical signs, symptoms, and surgical and histological results of adnexal torsion at different ages has not been thoroughly investigated.^6^ Knowledge of these factors may be helpful for improving the accuracy of diagnosing adnexal torsion before an operation and provide information for decision-making.

The treatment of adnexal torsion remains a challenging task with evolving techniques and practices in recent years.^7^ The majority of surgeons support a laparoscopic approach to conservative surgery (i.e., ovarian detorsion followed by ovarian cystectomy). In addition, if the surgeon is not skilled in laparoscopic surgery, an abdominal incision is also advised.

This study aimed to determine the differences in adnexal torsion among different age groups over 11 years in a single hospital setting. This information will hopefully allow for a more comprehensive understanding of adnexal torsion at different ages and provide information to improve the treatment strategies for patients with adnexal torsion.

## Material and methods

### Ethics

Before the research began, the study protocol was approved by the Institutional Review Board of Beijing Chao-Yang Hospital affiliated to Capital Medical University (IRB: 2024-department-494). A waiver of consent was granted because the data were obtained retrospectively from medical records. The study was conducted in accordance with the 1975 Declaration of Helsinki, as revised in 2013. All methods were performed in accordance with the relevant guidelines and regulations.

### Patients

The study population consisted of patients with a definitive diagnosis of adnexal torsion at surgery from January 2012 to November 2023 in the Obstetrics and Gynecology Department of our hospital. We excluded patients without torsion confirmed during surgery and patients who had no medical records. Data retrieved from the medical records of all patients included age, gravidity, parity, clinical presentations, medical history, ultrasonographic findings, laboratory data, surgical findings, and histopathological outcomes. The reporting of this study conforms to the STROBE guidelines.^8^

### Statistical analysis

In a descriptive analysis, categorical data are shown using frequencies and percentages. Continuous variables with a normal distribution are presented as the mean ± standard deviation. Median values and ranges are used to show variables that were not normally distributed. In an inferential analysis, categorical variables were compared between the groups with the chi square test or Fisher’s exact test (when expectancy was <5). Continuous variables were compared between the groups using the independent t-test or the Mann–Whitney test (if a normal distribution was found, the independent t-test was used). The distribution shape was mainly determined by a histogram. P < 0.05 was considered significant. GraphPad Prism 7.0 software (GraphPad Software, La Jolla, CA, USA) was used for all statistical analyses.

## Results

**Figure 1.**
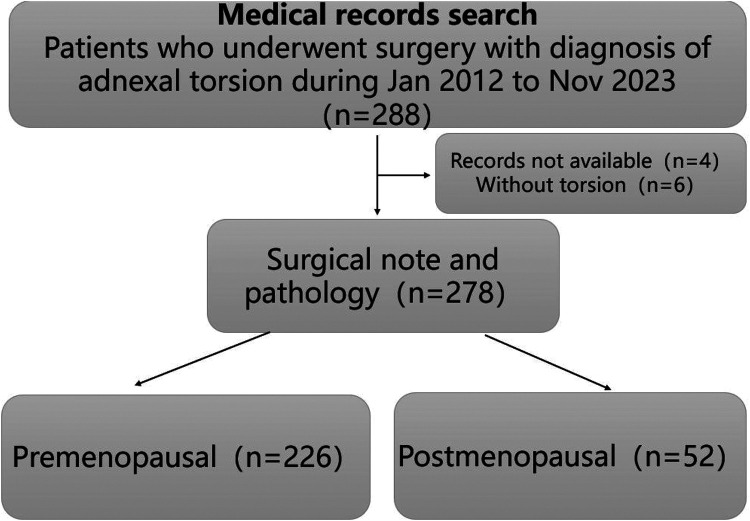
Flow chart of the study.

A total of 278 patients with adnexal torsion were included in the study. The patients were classified into two groups of 226 (81.3%) premenopausal women and 52 (18.7%) postmenopausal women according to their age at presentation (Figure 1). Among the premenopausal women, 24 (10.6%) were treated during pregnancy.

The median age of the patients was 31 years (19–53 years) in the premenopausal group and 64 years (49–89 years) in the postmenopausal group. The interval between the beginning of pain to admission in the postmenopausal group was significantly longer than that in the premenopausal group (P < 0.0001). The visual analogue score of the degree of pain at presentation was not significantly different between the groups. The postmenopausal group had a larger adnexal lesion size (P = 0.02), longer operation time (P = 0.0002), and greater blood loss during surgery (P < 0.0001) than the premenopausal group (Table 1).

**Table 1.** Clinical and operative characteristics of the two groups.

Characteristics	Premenopausal group (n = 226)	Postmenopausal group (n = 52)	P
Age (years)	31.97 ± 0.53	65.81 ± 1.56	<0.0001
Degree of pain	5.14 ± 0.14	4.71 ± 0.35	0.20
Interval of time from the onset of pain to hospital admission (hours)	34.43 ± 7.55	40.98 ± 8.24	<^&^0.0001
Operative time (minutes)	96.71 ± 3.30	133.3 ± 12.77	0.0003
Blood loss during surgery (mL)	38.54 ± 4.24	112.3 ± 32.48	<0.0001
Degree of torsion (°)	596.9 ± 20.53	602.9 ± 44.15	0.90
Size of the adnexal lesion (cm)	9.33 ± 0.25	10.82 ± 0.71	0.02
Hospital stay (days)	5.99 ± 0.22	10.25 ± 0.62	<0.0001

Data are the mean ± standard deviation.

& F test.

The suspected diagnostic rate using ultrasound in the premenopausal group was higher than that in the postmenopausal group (P < 0.0001) (Table 2). Abdominal pain occurred in 275 (98.9%) patients, nausea in 182 (65.5%), and vomiting in 141 (50.7%). No significant difference in any of these symptoms was found between the two groups. The incidence of a known adnexal mass or cyst was 28.4% (79/278), with no significant difference between the groups.

**Table 2.** Clinical symptoms and ultrasound characteristics of the two groups.

Variables	Presence/absence or details	Premenopausal group, n (%)(n = 226)	Postmenopausal group, n (%)(n = 52)	P
Nausea	+	149 (65.9)	33 (63.5)	0.75
	−	77 (34.1)	19 (36.5)	
Vomiting	+	112 (49.6)	29 (55.8)	0.45
	−	114 (50.4)	23 (44.2)	
Known cyst	+	63 (27.9)	16 (30.8)	0.73
	−	163 (72.1)	36 (69.2)	
US diagnosis	+	223 (98.7)	43 (82.7)	<0.0001
	−	3 (1.3)	9 (17.3)	
US flow	Decreased	42 (18.6)	9 (17.3)	0.39
	Disappeared	148 (65.5)	29 (55.8)	
	Normal	8 (3.5)	2 (3.8)	
	Other	28 (12.4)	12 (23.1)	

US, ultrasound.

Regarding the location of lesions, 114 (41.0%) lesions were on the left, 163 (58.6%) were on the right, and 1 (0.4%) was bilateral, with no significant difference between the two groups (Table 3). Regarding the type of procedure, the premenopausal group underwent significantly more conservative procedures, including cystectomy, resection of a fallopian cyst, and salpingectomy (113, 6, and 13 cases, respectively), than the postmenopausal group (P < 0.0001). The premenopausal group underwent significantly more procedures by laparoscopy than the postmenopausal group (P = 0.0002). However, the postmenopausal group underwent significantly more extensive procedures, such as hysterectomy (27 cases, 51.9%) and single or bilateral salpingo-oophorectomy (25 cases, 48.1%), than the premenopausal group (P < 0.001).

**Table 3.** Treatment and procedures in the two groups.

Variables	Details	Premenopausal group, n (%)(n = 226)	Postmenopausal group, n (%)(n = 52	P
Side	Left	94 (41.6)	20 (38.5)	0.76
	Right	131 (58.0)	32 (61.5)	
	Bilateral	1 (0.4)	0 (0)	
Treatment	Laparotomy	90 (39.8)	36 (69.2)	0.0002
	Laparoscopy	136 (60.2)	16 (30.8)	
Hysterectomy	Yes	8 (3.5)	27 (51.9)	<0.0001
	No	218 (96.5)	25 (48.1)	
Conservative procedure	Yes	132 (58.4)	0 (0)	<0.0001
	No	94 (41.6)	52 (100)	

Benign tumors were the most common pathological finding followed by tubal pathology in the two groups. As expected, functional cysts were only found in premenopausal women. The malignancy rate was similar between the two groups (Table 4).

**Table 4.** Histological findings of surgical specimens.

	Premenopausal group(n = 226)	Postmenopausal group(n = 52)	P value
Tubal/para-tubal pathology	29 (12.8)	3 (5.8)	0.23
Functional ovarian cyst	19 (8.4)	0	0.03
No pathology	16 (7.1)	2 (3.8)	0.54
Benign tumor	152 (67.3)	45 (86.5)	0.02
Borderline tumor	7 (3.1)	0	0.35
Malignancy	3 (1.3)	2 (3.8)	0.24

Data are n (%).

## Discussion

Our retrospective study showed that premenopausal and postmenopausal patients with adnexal torsion had similar main symptoms, but different surgical and histological results. The postmenopausal group had a longer interval to admission, a larger adnexal lesion, a longer operation time, greater blood loss, and underwent more extensive surgery than the premenopausal group. The incidence of malignancy was similar between the two groups.

Abdominal pain, nausea, and vomiting are the main symptoms of adnexal torsion. The previously reported incidence of nausea and vomiting in patients with adnexal torsion ranged from 52% to 70%.^3,9,10^ The incidence of typical symptoms in our study is similar to previously reported data,^11,12^ and these symptoms were similar among premenopausal and postmenopausal patients, with a high prevalence of abdominal pain and nausea or vomiting. All of these clinical features suggest that a diagnostic model of adnexal torsion based on clinical symptoms could be reliable.^13^

Although the presence of an adnexal mass or cyst could be a risk factor for adnexal torsion,^3^ the proportion of patients with a previously known adnexal cyst in this study was only 28.4% (79/278). Pregnancy is also a known risk factor for torsion, but it only occurred in 10.6% of our premenopausal patients. These findings suggest that there needs to be awareness of the importance of a regular health check-up for women.

Many researchers have proposed that sonographic criteria should be used to aid in the clinical diagnosis of torsion.^9,14,15^ The ultrasound characteristics of adnexal torsion include ovarian edema, adnexal mass, a change in Doppler flow, whirlpool sign, and pelvic fluid.^16,17^ Additionally, identifying adnexal torsion could be operator-dependent. In this study, preoperative ultrasound evaluation of local adnexal blood flow indicated that decreased or disappeared blood flow may be important for the evaluation of adnexal torsion, but adnexal torsion could not be excluded from a normal blood flow supply, which is consistent with another study.^14^

Regarding surgical results, the time interval from the onset of pain to emergency admission was longer in the postmenopausal group than in the premenopausal group. Additionally, the more extensive surgery in the postmenopausal group could have led to the longer operational time, more blood loss, and longer hospital stay. We also found that the diameter of the adnexal lesion in the postmenopausal group was larger than that in the premenopausal group, which is consistent with previous studies.^18^ The majority of torsion cases in both groups involved the right adnexa, which is similar to previous studies.^11^

The most common histological diagnosis of patients with adnexal torsion varies among studies.^5^ In this study, the most common pathology was a benign tumor of the ovaries. Malignancy was diagnosed in 3.8% of postmenopausal patients with adnexal torsion, which is a lower percentage than that found in two previous reports,^19,20^ but is similar to that in another report.^18^ In this study, the incidence of malignancy in premenopausal patients (1.3%) with adnexal torsion was low and is similar to that in other studies.^5,18,20,21^

## Strengths and limitations

Limitations of this study are its retrospective design and the lack of full documentation of the sonographic features of adnexal torsion because the sonographic description was limited to whether torsion was suspected. Laparoscopy is a minimally invasive, safe, and effective method in treating adnexal torsion, and should be considered in line with current practices. We need to respect the standards of good clinical practice in the 21st century. In addition, another limitation is that we only focused on cases with proven ovarian torsion by surgery. Therefore, we could not calculate the false-positive and false negative rates in the diagnosis of adnexal torsion.

## Conclusions

Clinical presentation of adnexal torsion is relatively similar in premenopausal and postmenopausal patients. Postmenopausal women have a longer interval of the onset of pain to hospital admission, a larger lesion size, a longer operation time, more blood loss, a longer hospital stay, and more extensive surgery than premenopausal women. Postmenopausal and premenopausal women with adnexal torsion have a similar incidence of malignancy.

## Data Availability

All data generated or analyzed during this study are included in this published article.

## Attestation statement

Data in the study have not been previously published or involved in other trials, and they will be made available to the editors of the journal for review or query upon request.
